# Palliative Cancer Care Ethics: Principles and Challenges in the Indian Setting

**DOI:** 10.4103/0973-1075.73639

**Published:** 2010

**Authors:** Tejaswi Mudigonda, Parvathi Mudigonda

**Affiliations:** National Institutes of Health, National Cancer Institute, Surgery Branch, Building 10 Hatfield CRC, Bethesda, MD 20892; 1 Leonard J. Chabert Medical Center, 1978 Industrial Boulevard, Houma, LA 70363

**Keywords:** Cancer care, Comprehensive cancer center, Palliative care, Palliative ethics

## Abstract

Palliative cancer treatment is a system of care that seeks to relieve suffering in patients with progressive cancer. Given the intractable symptoms with which certain malignancies manifest, palliative care offers a practical approach towards improving the patient’s quality of life. However, there are an array of ethical issues associated with this treatment strategy such as particular methods of pain relief, a reliable assessment of suffering, autonomy, and multi-specialist care. While these principles are important to increase and improve the network of palliative care, the resource-poor Indian environments present numerous barriers for these principles to be practically applied. As the infrastructure of comprehensive cancer centers develop, paralleled with an increase in training of palliative care professionals, significant improvements need to be made in order to elevate the status of palliative cancer care in India.

## INTRODUCTION

The inherent principle of palliative care is an integration of a solid support system with the primary focus to alleviate suffering and provide emotional and spiritual assistance. The ultimate goal is to establish a strong foundation of comprehension regarding the diagnosis and offer treatments that promote comfort and improve quality of life. Today, supportive and palliative care has been recognized as an important component of quality of life care for patients, particularly those with advanced or incurable disease such as cancer.[1] Despite modern treatment advances, approximately 50% of all cancer patients die from their disease, suggesting that for every second patient, the focus eventually has to shift from cure or life prolongation to palliation.[2] While palliative care may seem to offer an incredibly broad range of services, the goals of palliative treatment are concrete: relief from suffering, treatment of pain and other distressing symptoms, psychological and spiritual care, a support system to help the individual live as actively as possible, and a support system to sustain and rehabilitate the individual’s family.[3] Furthermore, it is important to acknowledge that the term “suffering” is a holistic one that adequately addresses all the elements of chronic illness and pain management.

Significant progress with palliative medicine continues to be made in India with the integration of palliative care in major cancer centers and the offering of fellowships in palliative medicine. Therefore, it is important to reinforce the continuing need for this alternative approach to cancer care in India and assess whether palliative care teams improve better quality of life outcomes. However, one of the barriers of ongoing palliative care development in India is the adopting of Western palliative care models, which are often irrelevant to the Indian setting. One aspect of Western palliative care that is particularly difficult to implement in the Indian context is the healthcare ethical standards by which palliative care centers are expected to uphold. The objective of this review is to describe the associated ethical issues of palliative cancer care and its challenges in the Indian setting.

## PALLIATIVE CANCER CARE MODEL

Part of the reasoning behind why advanced cancer patients receive inadequate care can be attributed to conventional medical philosophy that has its focus on curing illness and prolonging life, rather than improving the quality of life and relieving suffering. Traditionally, medical care has been articulated as having two mutually exclusive goals: either to cure disease and thereby prolong life or to provide comfort care.[4] The decision to provide comfort care enters the equation of treatment usually after life-prolonging treatment has been implemented (and often ineffectually cycled and repeated); only then does the idea of providing comfort care begin to fully develop, to say, after all other options have been exhausted.

**Figure 1:**
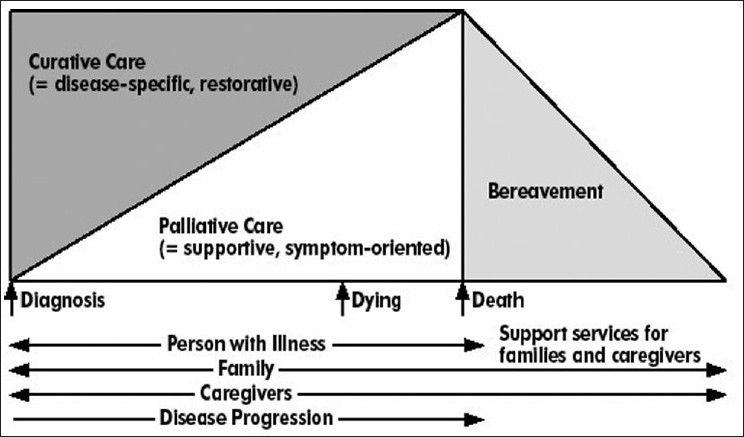
Model of palliative cancer care; Adapted from Ferris *et al*.

The understanding that palliative care is most effective when incorporated early in oncology care can lead to the development of the comprehensive cancer care model, which integrates palliative care along with anticancer therapy from the time of diagnosis. The American Society of Clinical Oncology has described responsibilities for oncologists to care for their patients in a continuum that extends from the moment of diagnosis throughout the course of the illness. In addition to appropriate anti-cancer treatment, this includes symptom control and psychosocial support during all phases of care, including those during the last phase of life.[1,5] The acceptable model in Figure 1 illustrates that clearly, cancer care is no longer a model of cure versus comfort care.[5]

## PALLIATIVE CANCER CENTERS IN INDIA

Fortunately, over the last 30 years, there are initiatives in a growing number of cancer centers that have implemented palliative programs. Palliative care in India is believed to have been originated in Kerala in 1986 with the establishment of a pain clinic in the Regional Cancer Center of Trivandrum, the capital of Kerala.[6] Since then, one recent 2008 review by McDermott *et al*,.[7] identified 138 hospice and palliative care services in 16 states and territories. For resource-poor settings, the efficacy of palliative care implementation has been driven by the synergistic effort of motivation and the use of local resources.[8] The Kerala network, called “Neighborhood Network in Palliative Care,” for example, has more than 60 units covering a population of >12 million and is one of the largest networks in the world.[9] In April 2008, Kerala became the first state in India to announce a palliative care policy.

However, cancer continues to remain an emerging public health problem in India, with nearly 0.9 million cases reported each year.[10] Palliative care in India is still at an early stage of development and faces numerous problems. Rajagopal *et al*.[11] critique the limitations of palliative care in India due to lack of access to opioids, inexpensive drugs, and psychosocial needs. The opinion among experts seems to vary about which approach to palliation is best. Some recommend improving infrastructure for palliative care (i.e. increase training, opening more hospices), while other suggest a cost-effective approach that best ensures affordability, availability, and coverage of more oral morphine.[12] Further efforts reflected in the Kerala model should pave for successful implementation of palliative care in India.

## PALLIATIVE CANCER ETHICS AND CHALLENGES

Provided this backdrop of palliative care in India, it is important to address the difficulty of imitating Western models of palliative care. In general, palliative cancer care has become a requisite for physicians while formulating a tailored plan of patient care. In the United States, the Supreme Court has voiced unequivocal support for adequate pain relief; and palliative medicine has become an area of expertise in its own right.[13] These developments prompt a review of some of the central ethical issues particular to palliative care. These issues such as relief of pain and suffering, autonomy and consent, and multi-specialist care, are important points of consideration for all physicians caring for patients regardless of the cause of their suffering and whether or not these physicians are specialists in palliative medicine or not.[14] At the same time, the Indian palliative care environment presents numerous challenges to these Western ethical principles of palliative care.

The first issue addresses the relief of pain and suffering. The availability, accessibility, and effectiveness of modern methods of pain control make it a moral mandate for every physician to be knowledgeable in the use of analgesics. It is estimated that less than 3% of India’s cancer patients have access to adequate pain relief.[15] Inadequate attention to pain relief is tantamount to moral and legal malpractice and is a violation of the principle of beneficence. Despite this, many physicians still lack an integrated comprehensive approach to pain management, which culminates to a fastidious use of analgesics. One cannot have discourse on pain management and exclude the ethical dilemma that the doses of analgesics sufficient to relieve some forms of chronic pain might hasten death (which sometimes may be an intended effect). It is permissible without fear of repercussion if the conditions of the “rule of double effect” are observed.[16] From a legal perspective, it is the physician’s obligation to provide adequate pain relief, while banning the practice of physician-assisted suicide.[17] In this context, palliative medication ought to be a constitutional right and that there is ethical and legal sanction for the use of whatever doses of narcotics are necessary so long as death is not directly intended.

However, there are numerous barriers to the assessment of relief of pain and suffering in Indian patients although physicians may have intent to do no harm. Developing countries such as India generally view the illicit drug epidemic in Western countries with caution and fear that opioid availability in India will lead to drug abuse.[18] The medicinal use of opioids such as morphine is highly regulated by the Indian Narcotic Drugs and Psychotropic Substances Act (NDPS), and to dispense morphine to patients the hospitals must be registered with the government and adhere to a set procedure.[10] Despite some success at increasing availability, progress is slow and opioid accessibility continues to remain a constant problem for the providers of palliative care in India.

The second issue deals with autonomy and consent. As with any program of treatment, consent for palliative care must be obtained from competent patients and cannot be assumed. Full disclosure is requisite so that the patient realizes he or she will be cared for by a multidisciplinary team of physician specialists (i.e. oncologists), nurses, social workers, pastoral care counselors, physical therapists, pain specialists, psychologists, and psychiatrists. However, part of the problem in India arises when vulnerable patients receiving palliative care become susceptible to suggestions about unorthodox treatment. Many Indians already choose so-called alternative medicines (i.e. ayurvedic, Siddha medicine, herbal medicine, homeopathic, etc.) alongside conventional allopathic medicine.[19] Such individuals are increasingly drawn to such treatment when conventional curative therapy fails.[20] Vaidya *et al*,.[21] state that Ayurvedic formulations have effectively controlled the side effects of chemotherapy. Siddha medicine, which supports the supernatural powers of healing, and herbal medicine are also very common elements in Indian alternative medicine. Especially in remote parts of the country, patients who live below the poverty line cannot afford palliative services and are compelled to try these alternative therapies.[6,12] Physicians who consider such treatment unethical ought to be free not to participate and should respectfully explain to the patient for their reasons to continue with such methods.

The third issue deals with the ethics of being treated by a multi-specialist palliative care team. As aforementioned, palliative cancer care comprises of various specialists collectively working to meet the needs of terminally ill patients. By the nature of cancer care, physicians are frequently exposed to patients and families with multiple, concurrent issues (physical, psychological, social, spiritual, etc.). To provide a secondary level of palliative cancer care in addition to the team of general practitioners, physicians require more knowledge and skill to manage complex situations and interactions with other physicians and patients. A review by Hearn *et al*,.[22] highlights that when compared to conventional care, there is evidence that specialist teams in palliative care improve satisfaction and identify and deal with more patient and family needs. Moreover, multi-professional approaches to palliative care reduce the overall cost of care by reducing the amount of time patients spend in acute hospital settings.[22]

However, the primary challenge to multi-specialist treatment approaches in India is the popularity of outpatient homecare programs and services. In one study of 33 palliative care clinics across Kerala, outpatient treatment with a supportive homecare service was adopted as the main mode of palliative care.[8] The homecare team primarily consists of trained nurses, family members, and even social workers, who travel in autorickshaws to visit terminally ill cancer patients and provide emotional support and advice for their symptom relief. Another example is the Bangalore Hospice Trust of the Indian Cancer Society, which has provided care for the terminally ill since 1994.[23] Therefore, instead of the inpatient multi-physician-based services rooted in Western care models, the convenient and cost-effective models of Indian palliative care often revolve around medical and psychosocial support delivered directly by nurses and family members. While the autonomy of the patient is seemingly undermined once the family and community become involved as the primary body of care, for many palliative cancer patients in India, this may be the only treatment option that is both practical and cost-effective. Furthermore, by reducing a broad specialist team approach to a limited homecare setting, this should not be perceived as a weakening of healthcare ethic and patient care.

Overall, in addition to providing better clinical outcomes, palliative medicine sets up a moral foundation whose methodology can become an ideology wherein there is only one morally right way to die. In doing so, the field medicalizes and professionalizes a process that depends as much on personal commitment to a friendly interest in the patient as it does on technical expertise. In the hospital and cancer center settings, oncologists have a responsibility to recognize when the benefits of treatment with chemotherapy or radiation have reached their limit so as not to delay comfort and palliative care unnecessarily. In this respect, it would be beneficial if oncologists were to spend time during their training in hospice or palliative care. Oncologists understandably are oriented to aggressive chemotherapy, and such treatments can be detrimental and even futile to the patient’s quality of life. From the patient’s point of view, oncologists must recognize their own biases in treating fatally ill patients. Communicating with patients is a core skill of palliative medicine, so it is important for oncologists to coordinate their patients’ care because the good of the patient demands it.[14]

## CONCLUSION AND FUTURE OUTLOOK

The goal of palliative care should continue to focus on the relief suffering and the improvement of the quality of life for patients with advanced illnesses such as cancer. It is based on an interdisciplinary approach that is offered simultaneously with other appropriate medical therapies and involves close monitoring of the emotional, spiritual, and practical needs and goals of patients and of their family members. Although several cancer centers today now have a palliative care program, significant gaps and delays in the delivery of care still remain.

Palliative cancer care is still an emerging and naïve healthcare context for both Indian patients and physicians. Efforts have only been started at the city or state level and there has been limited nationwide coordination of palliative care strategy. One survey comparing the level of awareness of palliative cancer amongst medical interns versus medical students at Kasturba Medical College revealed no improvement and relatively low levels of understanding of palliative care.[24] Such results only reflect inadequacies not only in government and patient populations but in the training academic institutions themselves. While there is the desire to imitate only Western models of palliation, the limitations in the Indian healthcare setting necessitate a future model of care adapted to the challenges of the Indian economy, government, and patient cohort.
